# Novel role of STRAP in progression and metastasis of colorectal cancer through Wnt/β-catenin signaling

**DOI:** 10.18632/oncotarget.7532

**Published:** 2016-02-20

**Authors:** Guandou Yuan, Bixiang Zhang, Shanzhong Yang, Lin Jin, Arunima Datta, Sejong Bae, Xiaoping Chen, Pran K Datta

**Affiliations:** ^1^ Division of Hematology and Oncology, Department of Medicine, UAB Comprehensive Cancer Center, University of Alabama at Birmingham, Birmingham, AL, USA; ^2^ Birmingham Veterans Affairs Medical Center, Birmingham, AL, USA; ^3^ Hepatic Surgery Center, Tongji Hospital, Tongji Medical College, Huazhong University of Science and Technology, Wuhan, China; ^4^ Division of Preventive Medicine, University of Alabama at Birmingham, Birmingham, AL, USA

**Keywords:** STRAP, β-catenin, CRC, metastasis, tissue microarray

## Abstract

Serine-Threonine Kinase Receptor-Associated Protein (STRAP) interacts with a variety of proteins and influences a wide range of cellular processes. Aberrant activation of Wnt/β-catenin signaling has been implicated in the development of colorectal cancer (CRC). Here, we show the molecular mechanism by which STRAP induces CRC metastasis by promoting β-catenin signaling through its stabilization. We have genetically engineered a series of murine and human CRC and lung cancer cell lines to investigate the effects of STRAP on cell migration and invasion *in vitro*, and on tumorigenicity and metastasis *in vivo*. Downregulation of STRAP inhibits invasion, tumorigenicity, and metastasis of CRC cells. Mechanistically, STRAP binds with GSK-3β and reduces the phosphorylation, ubiquitylation, and degradation of β-catenin through preventing its binding to the destruction complex. This leads to an inhibition of Wnt/β-catenin signaling and reduction in the expression of downstream targets, such as Cyclin D1, matrix metalloproteinases 2 and 9, and ß-TrCP. In human CRC specimens, higher STRAP expression correlates significantly with β-catenin expression with increased nuclear levels (*R* =0.696, *p* < .0001, *n* =128). Together, these results suggest that STRAP increases invasion and metastasis of CRC partly through inhibiting ubiquitin-dependent degradation of β-catenin and promoting Wnt/β-catenin signaling.

## INTRODUCTION

Wnt/β-catenin signaling plays a pivotal role in many human malignancies, especially in colorectal cancers (CRC) [1]. Even though the general mechanisms for Wnt/β-catenin signaling have been well established, some new components of the signaling pathway have still been identified, like WTX in Wilms tumor [2] and RACK1 in gastric tumor [3] were identified to interact within the destruction complex to modulate this signaling. Furthermore, β-catenin signaling can also be mediated by Wnt-independent signaling, such as EGFR [4], AKT [5] and JNK [6] etc. Although it has been reported that more than 80% of CRC have APC truncation/mutation or β-catenin mutation, both of which can activate the Wnt/β-catenin signaling during colorectal cancer development [7–9], Wnt/β-catenin signaling is also modulated through various other mechanisms in cancer, including crosstalk with other altered signaling pathways [7]. However, little is known about the role of this crosstalk in contributing to the hyperactivation of Wnt/ß-catenin signaling in CRC.

Serine-Threonine Kinase Receptor-Associated Protein (STRAP) is a WD40 domain-containing protein [10] that facilitates specific protein-protein interactions, sometimes leading to multi-protein complexes. We identified STRAP as a negative regulator of TGF-β signaling through the interaction with TGF-β receptors and Smad7 [10, 11]. Our previous study showed that STRAP is upregulated both in colon and lung carcinomas and promote tumorigenicity [12]. Subsequently, we have reported that STRAP can modulate EWS function in a TGF-β-independent manner [13]. STRAP has also been shown to regulate other multiple signaling pathways through direct physical interaction with other proteins, like PDK1 [14], NM23-H1 [15], ASK1 [16], and Sp1 [17] etc. Together, these findings suggest that STRAP functions as an oncogene through functional interaction with other signaling pathways. However, nothing is known about the role of STRAP in regulating Wnt/β-catenin signaling in colorectal cancer.

Our present study shows that knockdown of STRAP reduces CRC cell invasion and metastasis *in vitro* and *in vivo*. In an attempt to understand the mechanism, we have observed that STRAP stabilizes β-catenin by inhibiting its ubiquitin-dependent degradation, thus resulting in the inhibition of the expression of its downstream target genes. Most interestingly, in support of these results we have observed that both STRAP and β-catenin is co-upregulated in high percent of human CRC (*R* = 0.696, *p* < .0001, *n* =128). Thus, our results provide evidence of how STRAP is involved in the contribution to CRC development and progression by a unique mechanism.

## RESULTS

### Effect of downregulation of STRAP on migration, invasion and tumorigenicity in CRC cell lines

**Figure 1:**
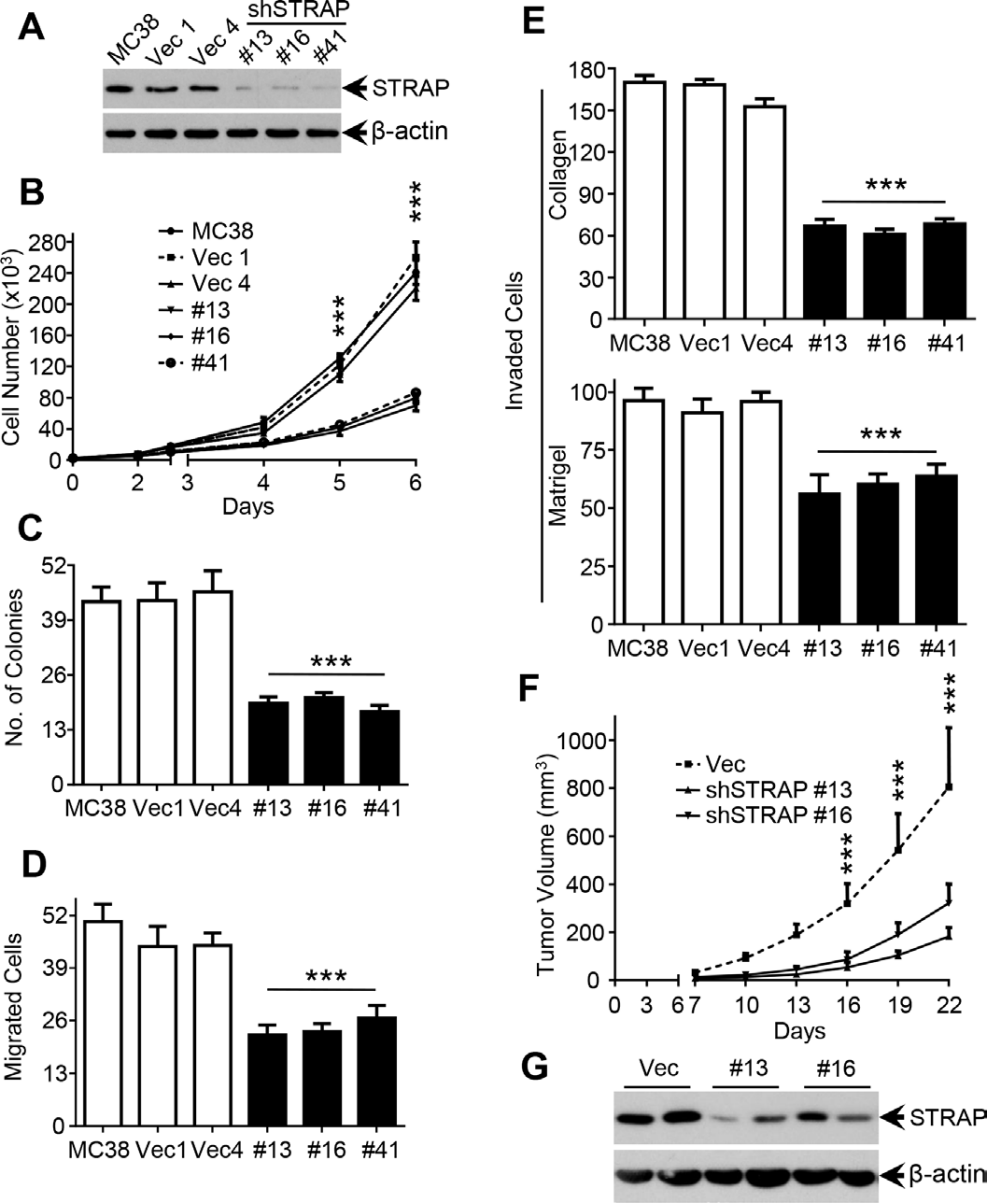
Role of STRAP on migration, invasion and tumorigenicity in CRC cell lines (**A**) Expression of STRAP in MC38 stable clones after transfection of STRAP shRNA was examined by western blotting. β-actin was used as loading control. (**B**) Cell counting assay. MC38 stable clonal cells with STRAP knockdown and parental and vector control cells were cultured for a total of 6 days. Cells were counted everyday for 5 days from the third day after the cells were seeded and the cell numbers are plotted. Individual data points are mean ± S.D. of triplicate determinations. ***P < .001. (**C**) Soft agarose assay. MC38 cells were cultured in 0.4% sea plague agarose for 14 days. Number of colonies is counted and shown as mean ± S.D. of triplicate wells. ***P < .001. (**D**) Cell migration assay. MC38 cells were allowed to migrate through collagen coated transwells for 6 h. Then the migrated cell were fixed and stained. Six random high power fields in each well were counted. Each data point represents mean ± S.D. from three wells. ***P < .001. (**E**) Cell invasion assay. MC38 cells were allowed to pass through a collagen barrier (top) or a matrigel layer (bottom) in the transwell chambers. Then the invaded cells were fixed and stained. Six random high power fields in each well were counted. Each data point represents mean ± S.D. from three wells. ***P < .001. (**F**) Suppression of the tumorigenicity of MC38 *in vivo* by knockdown of STRAP. Results are presented as mean ± S.D. of the tumor volume (top). ***P < .001. (**G**) The expression of STRAP in subcutaneous tumors was analyzed by western blotting.

Our previous study has shown that STRAP is upregulated in colon carcinoma and upregulation of STRAP in human cancers may provide growth advantage to tumor cells via TGF-β-dependent and TGF-β-independent mechanisms [12]. To investigate the role of STRAP on invasion and metastasis in CRC, we stably knocked down STRAP in murine colon carcinoma cell lines MC38 and CT26 as determined by western blotting (Figure 1A and Supplementary Figure S1A). To evaluate the effects of STRAP on tumorigenicity of CRC cells *in vitro*, we performed cell counting and soft agar assays. Downregulation of STRAP significantly inhibited CRC cell growth in liquid culture as well as in soft agar in both MC38 and CT26 cells (Figure 1B and 1C and Supplementary Figure S1B and S1C). To explore the role of STRAP knockdown on cell migration and invasion, we performed trans-well migration and invasion assays (through collagen and matrigel). As shown in Figure 1D and 1E and Supplementary Figure S1D, knockdown of STRAP in these two cell lines reduced cell migration and invasion. Next, we investigated the effects of STRAP on tumorigenicity of CRC cells *in vivo* using xenograft models. When compared with the vector control cells, downregulation of STRAP remarkably inhibited tumor growth in syngeneic mice (Figure 1F and Supplementary Figure S1E). Lower expression of STRAP in tumors derived from knockdown clones was maintained (Figure 1G and Supplementary Figure S1F). Together, these results suggest that STRAP promotes tumorigenic behavior of CRC cells *in vitro* and *in vivo*.

### Role of STRAP on regulating β-catenin expression and signaling in CRC cell lines

**Figure 2:**
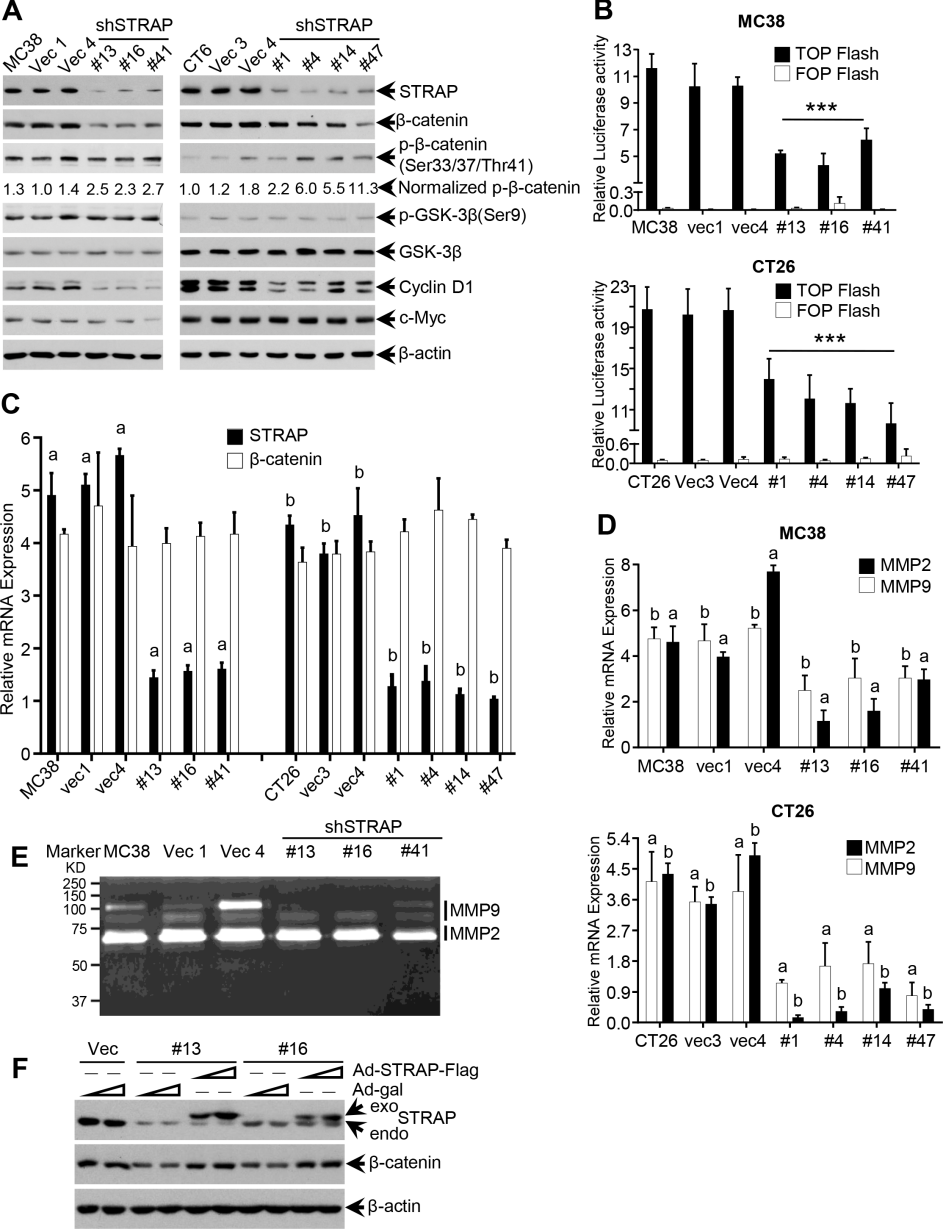
Regulation of β-catenin expression and signaling by STRAP in CRC cell lines (**A**) Some important components of Wnt/β-catenin signaling were tested by western blotting with the lysates from MC38 and CT26 stable clones. (**B**) The transcriptional activity of Wnt/β-catenin signaling was inhibited by STRAP knockdown in both MC38 (top) and CT26 cells (bottom). TOP Flash or FOP Flash and β-galactosidase plasmids were transfected into MC38 and CT26 stable clone cells. After 48 h of transfection, luciferase activity was determined and normalized to β-galactosidase activity. The luciferase values were shown as mean ± S.D. from triplicate wells. ***P < .001. (**C**) The expression of STRAP and β-catenin mRNAs in STRAP knock down clones from MC38 and CT26 cells was determined by real-time qRT-PCR. a & b, *P* < .001. (**D**) The expression of MMP2 and MMP9 in STRAP knock down clones was examined by Real-Time qRT-PCR. a & b, *P* < .001. (**E**) The activation of MMP2 and MMP9 in STRAP knock down clones was detected by Zymography. (**F**) Replication deficient adenoviruses (RDA), that are able to transiently express Flag-tagged STRAP, were infected into MC38 stable clones. After 60 hours of incubation, the cells were harvested. The expression of STRAP and β-catenin was examined by western blotting.

To determine whether upregulation of STRAP in CRC regulates Wnt/β-catenin signaling, we first examined β-catenin protein expression in STRAP knockdown MC38 and CT26 clones by western blotting. β-catenin was significantly downregulated in knockdown clones when compared with that in control cells (Figure 2A). On the contrary, relative phosphorylation of β-catenin at Ser33/Ser37/Thr41, which initiates β-catenin ubiquitin-dependent degradation [18], was significantly increased. To detect whether STRAP can regulate β-catenin subcellular distribution, we examined β-catenin expression in different subcellular fraction. In concert with the total β-catenin, both cytoplasmic and nuclear β-catenin was decreased in stable clones (Supplementary Figure S2A and S2B). These findings prompted us to investigate whether STRAP can activate Wnt/β-catenin signaling by increasing β-catenin expression in CRC. We tested Wnt/β-catenin signaling activity using its signaling reporter TOP Flash, which contains three copies of an optimal TCF binding motif (CCTTTGATC), and FOP Flash as a negative control. Downregulation of STRAP significantly inhibited the activity of TOP Flash in both CRC cell lines when compared with vector controls (Figure 2B). To further validate this hypothesis, we tested the expression of Wnt/β-catenin signaling target genes, including Cyclin D1 [19], c-Myc [20], β-TrCP [21], MMP2, MMP7 and MMP9 [22, 23]. Cyclin D1 and β-TrCP level was reduced in STRAP knockdown clones from both cell lines (Figure 2A and Supplementary Figure S2C and S2D). However, there was not much difference in c-Myc expression that might be due to cancer type and/or cellular context. In addition, we didn't see any difference either in the level of GSK-3β or in its phosphorylation at Ser9 [24, 25]. Downregulation of STRAP inhibited the expression of MMP2 and MMP9 at the transcription level (Figure 2D) and their activity (Figure 2E), but not for MMP7 (data not shown). In an attempt to determine whether STRAP regulates β-catenin in transcriptional level, we did not see any difference in β-catenin mRNA level after STRAP knock down (Figure 2C) suggesting that STRAP promotes Wnt/β-catenin signaling through stabilizing β-catenin protein. To determine the specificity of this effect of STRAP, we performed the rescue experiment through infecting MC38 vector and STRAP knockdown clones by STRAP-Flag adenovirus or β-gal adenovirus. As shown in Figure 2F, β-catenin expression was restored when STRAP expression was rescued in stable knockdown clones. Together, downregulation of STRAP inhibits Wnt/β-catenin signaling by reducing β-catenin expression.

### Role of GSK-3β and Wnt3a on STRAP-induced stabilization of β-catenin

**Figure 3:**
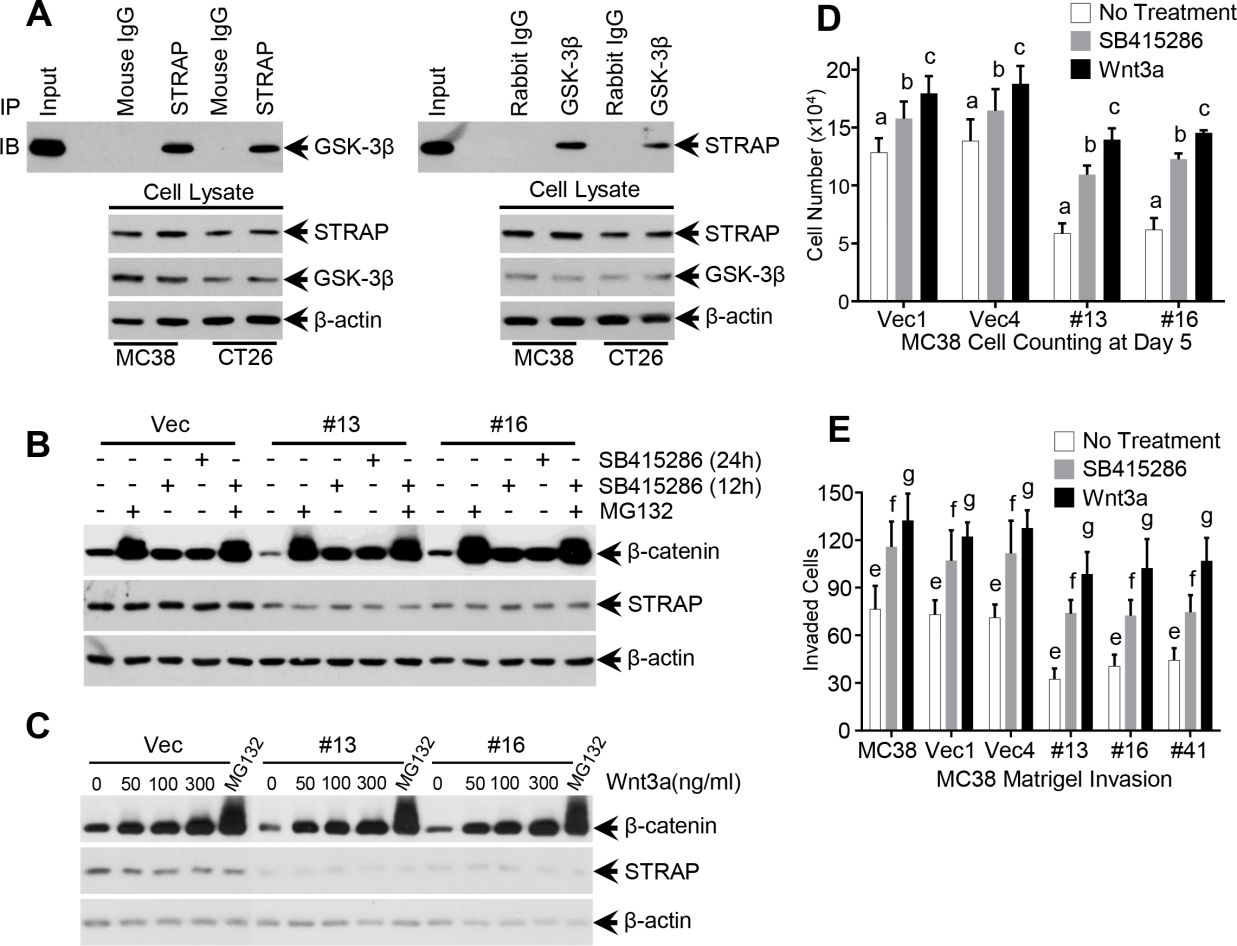
Role of GSK-3β inhibitors, proteasomal inhibitor and Wnt3a on STRAP-induced stabilization of β-catenin (**A**) Endogenous interaction between STRAP and GSK-3β in colon cancer cell lines. Lysates from MC38 and CT26 were subjected to immunoprecipitation using 1 ug of mouse anti-STRAP, rabbit anti- GSK-3β antibodies or corresponding IgG (negative control). Immunoprecipitated STRAP or GSK-3β was detected by western blotting. (**B**) MC38 stable clonal cells were treated with GSK-3β inhibitor SB415286 (25 uM, two time points) and proteasomal inhibitor MG132 (25 uM, 6 h). Then the lysates were subjected to western blotting for β-catenin and STRAP. (**C**) MC38 stable clonal cells were treated with Wnt3a (6 h) for different doses. Treatment with MG132 was used as positive control. Then the cells were harvested and subjected to western blotting for β-catenin and STRAP. (**D**) Growth inhibition in MC38 stable clones was partially abolished by treating with SB415286 and Wnt3a. MC38 stable clones with STRAP knockdown and parental and control vector cells were treated with 12.5 uM SB415286 or 50 ng/ml Wnt3a, and counted everyday for 5 days. The cell numbers of day 5 were shown. b & c, *P* < .01, a, *P* < .001. (**E**) Reduction in invasion of MC38 stable clones was partially rescued by treating with SB415286 and Wnt3a. MC38 cells were seeded on a thin layer of Matrigel. 9 hours later, after the cells settling down, the cells were treated with 12.5 uM SB415286 or 50 ng/ml Wnt3a for another 12 hours. The invaded cells were counted for six random high power fields in each well. Each data point represents mean ± S.D. from three wells. e, f, g, *P* < .001. These experiments were repeated three times.

We have previously shown that STRAP binds with GSK-3β when both proteins were overexpressed in 293T cells and GSK-3β inhibitors can reduce this binding [26]. To investigate the endogenous binding in CRC cell lines, we performed immunoprecipitaion assays with anti-GSK-3β antibody or anti-STRAP antibody using lysates from MC38 and CT26 cells. STRAP coprecipitated GSK-3β and vice versa, thus suggesting an endogenous interaction in CRC cell lines (Figure 3A). These findings prompted us to explore whether GSK-3β inhibitor and proteasomal inhibitor regulates the effect of STRAP on β-catenin expression. We treated MC38 cell clones with GSK-3β inhibitors LiCl (20 mM) and SB415286 (25 uM), and proteasomal inhibitor MG132 (25 uM), and then tested β-catenin expression and subcellular localization by western blotting and immunofluorescence, respectively. As shown in Figure 3B and Supplementary Figure 3, β-catenin was completely restored after treatment with GSK-3β inhibitors and MG132 when compared with vector control, although the basal β-catenin levels were lower in STRAP knockdown clones. These results further suggest that STRAP stabilizes β-catenin through interacting with GSK-3β. Next, to test whether Wnt ligand has any effect on STRAP-induced stabilization of β-catenin, we treated MC38 cell clones with increasing doses of Wnt3a and the expression of β-catenin was detected. β-catenin was upregulated similarly in a dose dependent manner in both vector and STRAP knockdown clones, suggesting that the effect of STRAP was superseded by Wnt3a (Figure 3C). To further evaluate the biological outcome of these effects, we performed cell counting and matrigel invasion assays with MC38 cell clones after treating with SB415286 or Wnt3a. SB415286 and Wnt3a promoted CRC cell growth (Figure 3D) and invasion (Figure 3E) in both vector control and knockdown clones when compared with corresponding no-treatment group. However, STRAP knockdown stable clones still showed lower cell growth (Figure 3D) and invasion (Figure 3E) when compared with vector control with the same treatments, suggesting that downregulation of STRAP in CRC cell lines inhibits cell growth and invasion partly through inhibiting Wnt/β-catenin signaling. These findings suggest that STRAP promotes cell growth and invasion in CRC through regulating β-catenin expression.

### Inhibition of ubiquitin-dependent degradation of β-catenin by STRAP

**Figure 4:**
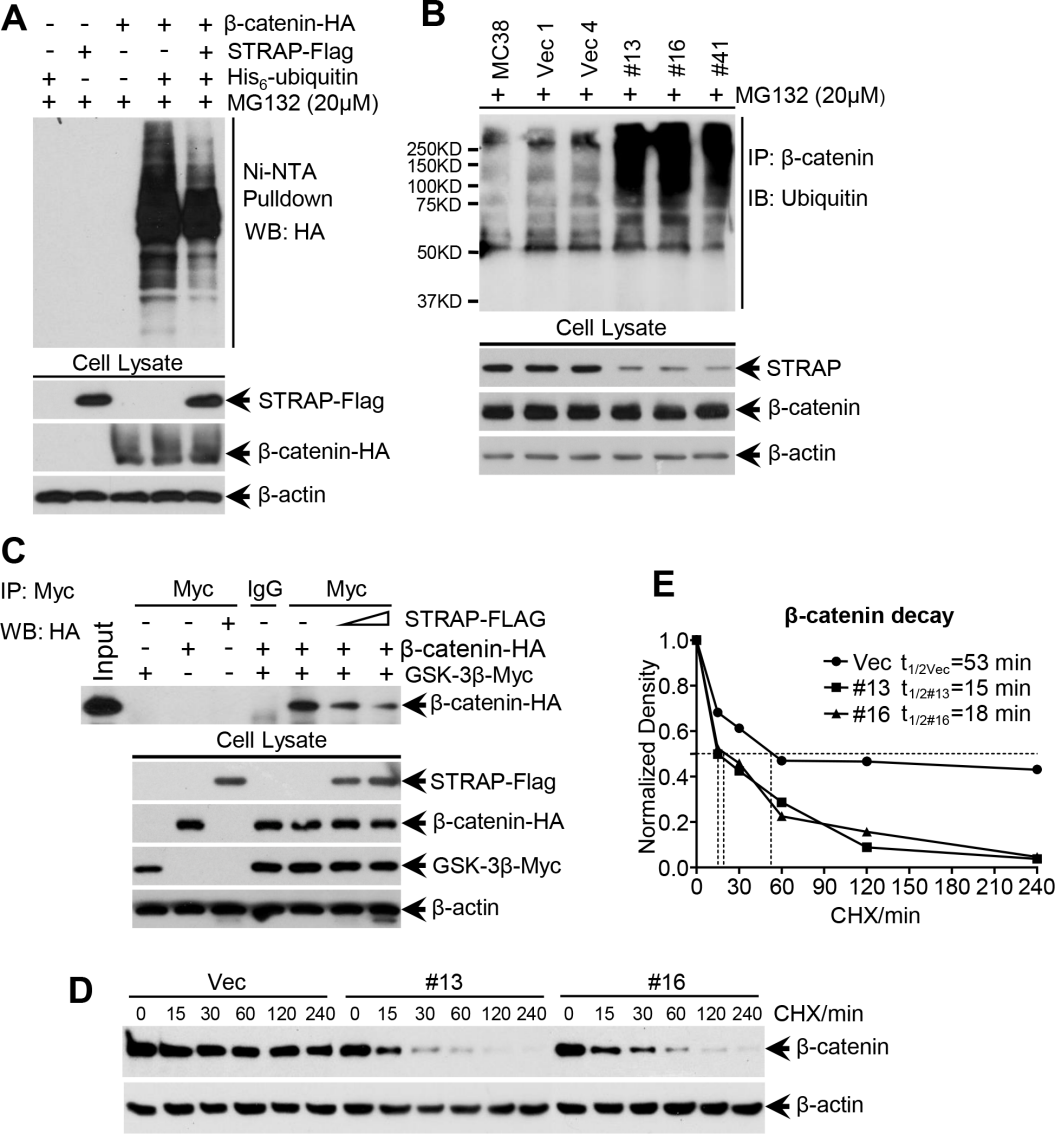
Effect of STRAP on ubiquitin-dependent degradation of β-catenin (**A**) Exogenous ubiquitin assay. 293T cells were transfected with HA-tagged β-catenin, His6-tagged ubiquitin and Flag-tagged STRAP in combinations as indicated. After treated with MG132, the cells were lysed in a modified lysis buffer as detailed in the Materials and Methods. Proteins tagged with His_6_-ubiquitin molecules were pulled down with Ni-NTA agarose beads. Eluted proteins were subjected to western blotting with anti-HA antibody to detect ubiquitinated β-catenin. Expression of the proteins was tested by western blotting. (**B**) Endogenous ubiquitin assay. The same amount of lysates from MC38 stable clones were harvested after treatment with MG132 for immunoprecipitation with anti-β-catenin antibody. The ubiquitinated β-catenin was evaluated with ubiquitin antibody by western blotting. (**C**) STRAP inhibits β-catenin binding to the destruction complex. 293T cells were transfected with β-catenin-HA, GSK-3β-myc and different doses of STRAP-Flag plasmids in combinations as indicated. The lysates were subjected to immunoprecipitation with c-myc antibody, and bound β-catenin was detected by western bloting with anti-HA antibody. Expression of the proteins was tested by western blotting. (**D**) and (**E**) STRAP inhibits the β-catenin protein degradation in MC38 stable clones. MC38 stable clones were treated with cycloheximide (CHX, 100 ug/ml) and harvested after the indicated time of treatment. β-catenin protein levels were analyzed by Western blotting (D). The density of β-catenin was normalized against β-actin and the relative density is presented (E). Half-life of β-catenin in different clones was calculated.

The above observations prompted us to investigate whether STRAP stabilizes β-catenin through inhibiting its ubiquitin-dependent degradation. To evaluate this hypothesis, we first performed exogenous β-catenin ubiquitin assay in 293T cells. Overexpression of STRAP reduced ubiquitylated β-catenin in the pulldown/western blot experiment (Figure 4A). For endogenous ubiquitin assay, we directly immunoprecipitated β-catenin and the ubiquitylated β-catenin was detected by anti-ubiquitin antibody. The ubiquitylated endogenous β-catenin level was much higher in STRAP knock down stable clones when compared with that in control cells (Figure 4B). These observations prompted us to presume that STRAP might block β-catenin binding to the destruction complex when binding with GSK-3β and Axin. To validate this hypothesis, we co-transfected β-catenin and GSK-3β plasmids into 293T cells with increasing doses of STRAP plasmid. GSK-3β was immunoprecipitated from the cell lysates, and the immune-complexes were analyzed by western blotting for β-catenin. STRAP inhibited β-catenin binding to its destruction complex in a dose dependent manner (Figure 4C). To further prove that STRAP can stabilize β-catenin, we treated MC38 cell clones with cycloheximide for different time points as indicated. We observed that knockdown of STRAP strongly promoted β-catenin degradation in stable clones when compared with that in vector clone and decreased the half-life of β-catenin (Figure 4D and 4E). These results suggest that STRAP stabilizes β-catenin by inhibiting its interaction with GSK-3β and subsequent ubiquitin-dependent degradation.

### Role of STRAP on CRC metastasis in an orthotopic model

**Figure 5:**
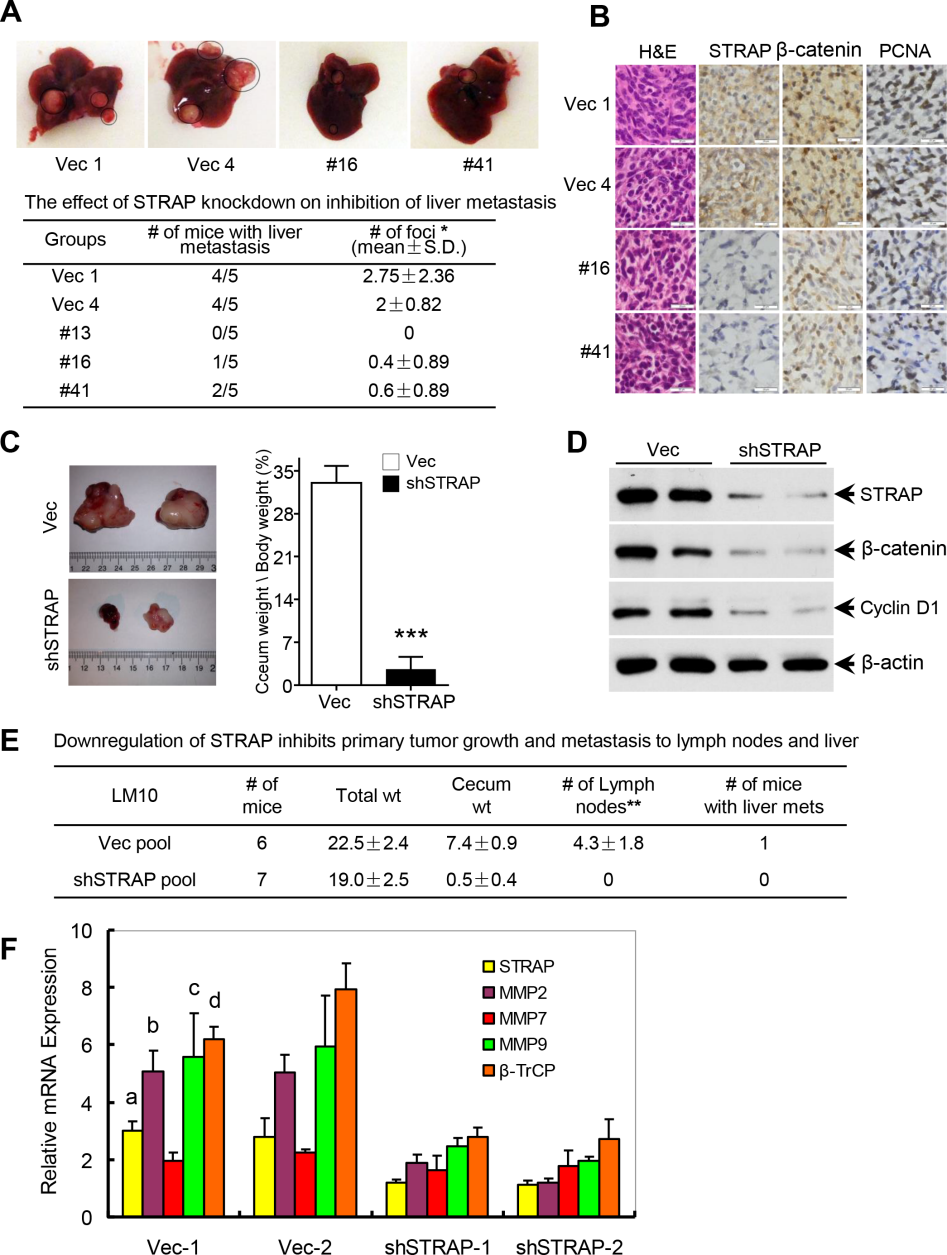
Role of STRAP on CRC metastasis in splenic injection and orthotopic models (**A**) MC38 stable clone cells were injected into spleens of C57BL/6 mice (*n* = 5, in each group). Three weeks after injection the mice were euthanized. Representative pictures of liver metastasis were shown and the foci of liver metastasis were assessed. *P < .05. (**B**) Paraffin sections from the liver metastases were immunostained for H & E, STRAP, β-catenin and PCNA. Calibration bars represent 20 μm. (**C**) In order to establish representative and reproducible animal models to study spontaneous metastasis of colorectal cancer, we generated a highly aggressive and metastatic mouse cell line (LM10) that was derived from MC38 parental cells after passing 10 cycles stepwise through splenic injection model of liver metastasis. LM10 cells were subserosally injected into the ceca of C57BL/6 mice for primary tumor growth and subsequent metastasis. Representative pictures of orthotopic tumor growth in the ceca were shown (left) and relative cecum weight/body weight was presented as mean ± S.D. (right). ***P < .001. (**D**) The expression of STRAP, β-catenin and Cyclin D1 in ceca tumors was assessed by western blotting. (**E**) The effects of STRAP knock down on inhibition of primary tumor growth and metastasis to lymph nodes and liver were presented as mean ± S.D. **P < .01. (**F**) The expression of β-catenin signaling downstream target genes β-TrCP, MMP2, MMP7 and MMP9 in the primary tumor tissues was analyzed by real time qRT-PCR. a, b, c & d *P* < .001, all compares between vector groups and shSTRAP groups.

To evaluate the biological function of STRAP stabilizing β-catenin in CRC, splenic and orthotopic cecum injection models of metastasis were performed. For splenic injection, MC38 and CT26 cells were injected into spleens of syngeneic C57BL/6 and Balb/c mice, respectively, to generate liver metastases. Downregulation of STRAP significantly reduced the rate of metastatic foci formation as well as their growth and spread in the liver in both cell lines (Figure 5A and Supplementary Figure S4A and S4B). Lower expression of STRAP in liver metastases derived from knockdown clones decreased the expressions of β-catenin, some of its target genes, and the proliferation marker PCNA when compared to those in vector control tumors (Figure 5B and Supplementary Figure S4C and S4D). For the orthotopic cecum injection model, we have generated a highly aggressive and metastatic mouse MC38-LM10 (LM10) cell line that was derived from MC38 cells after passing 10 cycles stepwise through splenic injection model of liver metastasis. LM10 cells were subserosally injected into the ceca of C57BL/6 mice to generate primary tumors and metastases. Downregulation of STRAP remarkably reduced primary tumor growth in ceca (Figure 5C), in which no mice had lymph node metastasis, but all vector control mice had lymph node metastases and one mouse had liver metastasis (Figure 5E). Lower expression of STRAP in tumors from knockdown cells resulted in the downregulation of β-catenin and its downstream targets Cyclin D1, β-TrCP, MMP2 and MMP9 (Figure 5D and 5F). Together, STRAP promotes CRC tumorigenicity and metastasis *in vivo* through regulating β-catenin expression and signaling, which is consistent with our *in vitro* observations.

### Effect of β-catenin mutation and APC truncation on STRAP induced stabilization of β-catenin

**Figure 6:**
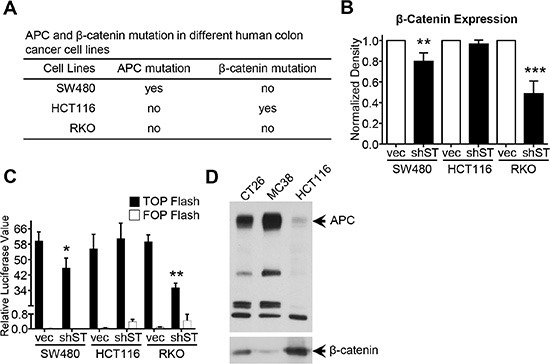
Role of β-catenin mutation and APC deletion on STRAP induced stabilization of β-catenin (**A**) The mutation status of APC and β-catenin in SW480, HCT116 and RKO was presented. (**B**) Lysates from SW480, HCT116 and RKO polyclones after knocking STRAP down were subjected to western blotting for STRAP, β-catenin. β-actin was used as loading control. Normalized expression of β-catenin from three independent experiments was presented as mean ± S.D. **P < .01, ***P < .001. (**C**) The transcriptional activity of Wnt/β-catenin signaling was detected in SW480, HCT116 and RKO polyclones after knocking STRAP down using TOP/FOP Flash reporter as described above. *P < .05, **P < .01. (**D**) The expression of wild type (wt) APC in MC38 and CT26 was detected by western blotting. The same amount of lysates from MC38, CT26 and HCT116 were subjected to western blotting. HCT116 was used as positive control for wt size of APC.

About 80% of CRC have APC truncation and about 10% of CRC bear β-catenin mutation, both of which can activate the Wnt/β-catenin signaling during progression [7–9]. To investigate the effect of these mutations on STRAP-induced stabilization of β-catenin, we chose three different human colon cancer cell lines, SW480, HCT116 and RKO having different mutational status in APC and β-catenin genes (Figure 6A). After the knockdown of STRAP, we did not see any effect on β-catenin protein stabilization in HCT116 cells, which has activating mutation at Ser45 of β-catenin. However, knockdown of STRAP significantly decreased β-catenin protein stabilization in the isogenic cell lines, SW480 (Figure 6B) and SW620 (Supplementary Figure S5A) having truncation at 1338aa of APC gene. In contrast, RKO cells with no mutation in β-catenin and APC gene showed 50% decrease in the level of β-catenin (Figure 6B). These results were further supported by TOP/FOP flash reporter luciferase assays in these cell lines. Downregulation of STRAP significantly inhibited the activity of TOP Flash in SW620 (Supplementary Figure S5B), SW480 and RKO cell lines (Figure 6C), but not in HCT116 cells. Interestingly, we also found that MC38 and CT26 had wt β-catenin (data not shown) and wt APC (Figure 6D), which further validate these observations. These results suggest that STRAP has no effect on stabilizing β-catenin in β-catenin mutated CRC cells, but has partial effect in APC truncated cells.

### Stabilization of β-catenin by STRAP in lung cancer

Recently, increasing evidences have suggested that Wnt/β-catenin signaling plays an important role in lung carcinogenesis, which has much less APC and β-catenin mutations unlike colon cancers [27]. Besides, our previous studies have shown that STRAP is up regulated in 78% of lung carcinomas [12]. All these evidences prompted us to investigate whether STRAP has any effect on stabilizing β-catenin in lung cancer. To validate this, we chose two non-small cell lung cancer (NSCLC) cell lines H460 and A549, both of which have wt APC and wt β-catenin. Interestingly, we observed similar results as in CRC cell lines, like knockdown of STRAP increased β-catenin phosphorylation, decreased the expression of β-catenin and Cyclin D1 and TOP Flash reporter activity (Supplementary Figure S6A-S6C). We did not observe any change in β-catenin mRNA level, whereas MMP2 mRNA was decreased in STRAP knockdown clones (Supplementary Figure S6D). These results further generalize the effects of STRAP on stabilizing β-catenin through inhibiting its ubiquitin-dependent degradation.

### Correlation between the expression of STRAP and β-catenin in colorectal cancer

**Figure 7:**
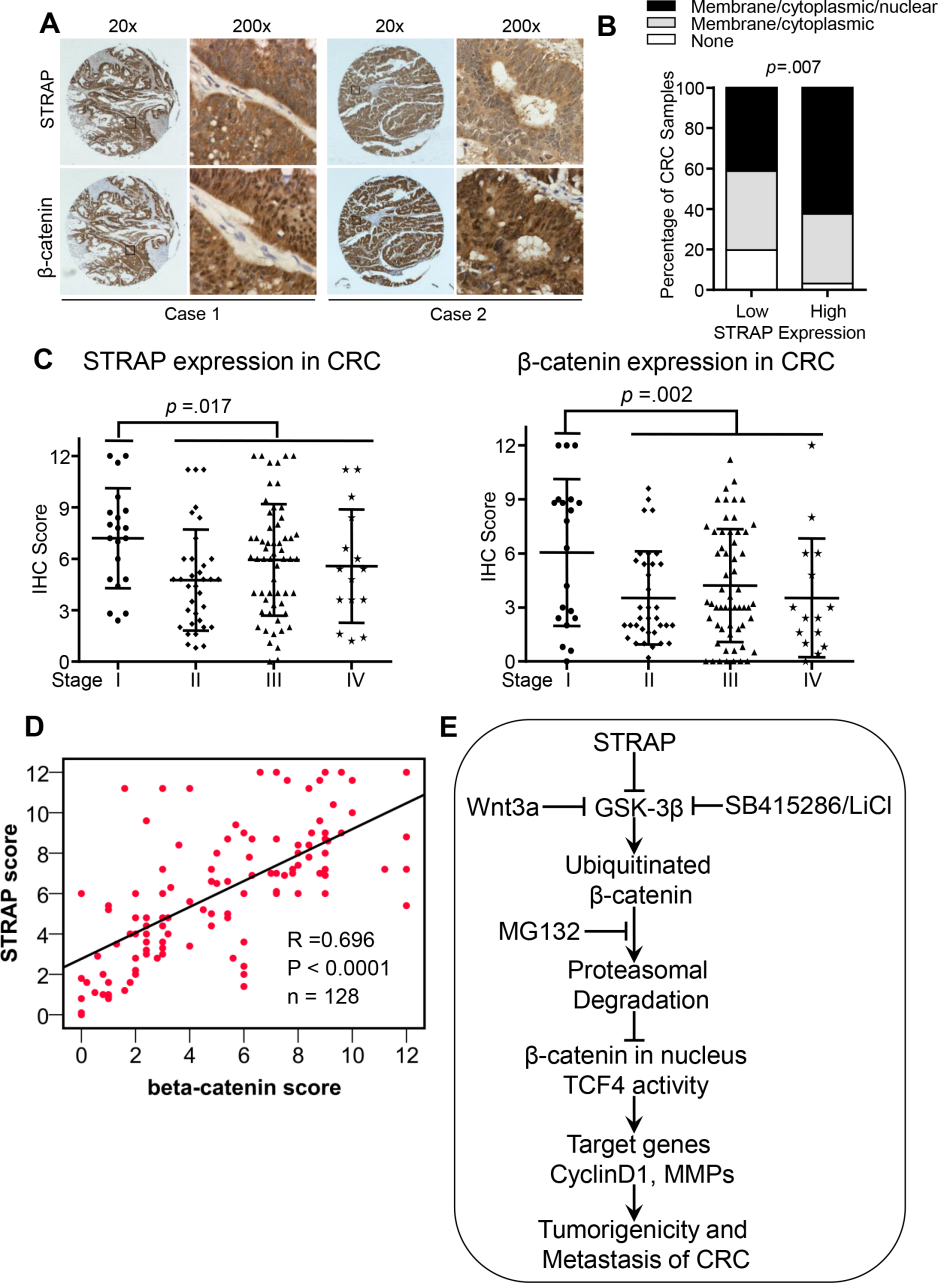
Correlation between the expression of STRAP and β-catenin in CRC Total 130 cases of CRC samples were immunostained with anti-STRAP and anti-β-catenin antibodies. The immunohistochemical evaluation for human colon cancer tissues for the expression of STRAP and β-catenin include the staining intensity and percentage of cells stained as described in Materials and Methods. (**A**) Representative pictures of immunostaining of STRAP and β-catenin in human CRC samples. (**B**) STRAP stabilizes β-catenin and increased β-catenin nuclear accumulation was observed in higher STRAP expression group. (**C**) The expression of STRAP and β-catenin in different AJCC stages of CRC was plotted. (**D**) The expression of STRAP and β-catenin in CRC patient samples were analyzed for correlation by spearman rank correlation coefficient analysis. *R* = 0.696, *p* < .0001, *n* =128. (**E**) Schematic model for STRAP modulating Wnt/β-catenin signaling in colorectal cancer. Upregulation of STRAP stabilizes β-catenin by inhibiting its ubiquitin-dependent degradation, and subsequently increases β-catenin/TCF4 transcriptional activity. These effects result in enhanced expression of β-catenin target genes, thus leading to the increase in tumorigenicity in CRC.

Based on the previous observations that the expression of STRAP is upregulated in human colorectal cancers [12, 28] and our findings that STRAP stabilizes β-catenin, we predicted that the expression levels of STRAP and β-catenin would be functionally correlated in colorectal cancers. To test this hypothesis, we immunostained for STRAP and β-catenin in serial sections of colon tissue microarrays (TMA) containing 130 CRC patient specimens. Consistent with previous reports, the expression of both STRAP [12, 28] and β-catenin [29] was upregulated in 76.9% and 71.2%, respectively (Supplementary Table S2). We also noticed that 50.8% cases had β-catenin nuclear accumulation, which is known to be associated with CRC prognosis [30]. Interestingly, we also observed higher nuclear accumulation of β-catenin in STRAP high expression group compared to low expression group, when STRAP was mostly localized in the cytoplasm (Figure 7A and 7B). A statistically significant positive correlation was observed between the expressions of STRAP and β-catenin in these specimens as shown in Figure 7D (R = 0.696, *p* < .0001, *n* =128). Furthermore, we found that the expression of both STRAP and β-catenin were much higher in AJCC stage I than that in other stages (Figure 7C), indicating that both STRAP and β-catenin may function in the early stage of CRC tumorigenesis. Together, these observations indicate that there is a highly significant correlation between the expression of STRAP and the expression and nuclear localization of β-catenin in CRC. This further validates our *in vitro* findings that STRAP stabilizes β-catenin by reducing its ubiquitin-dependent degradation.

## DISCUSSION

Our previous studies have shown that STRAP is upregulated in CRC and lung cancer, and can provide growth advantage to tumor cells via TGF-β-dependent and -independent mechanisms [12]. In this study, we have explored its new invasive and metastatic functions in CRC through a novel mechanism. STRAP activates Wnt/β-catenin signaling and regulates downstream target genes through stabilizing β-catenin protein. Higher expression of these two proteins in the early stage of CRC progression and increased nuclear accumulation of β-catenin in tumors with higher STRAP expression suggest their cooperative role in the development and progression of CRC.

In this study, we have noted that expression of STRAP significantly reduced binding of β-catenin to the destruction complex probably through steric hindrance (Figure 4C) and inhibited its subsequent ubiquitylation (Figure 4A and 4B). Both Wnt stimulation and inhibition of GSK-3β activate Wnt/β-catenin signaling in STRAP knock down cells through its stabilization, suggesting that the effect of STRAP was superseded by these agents. These findings provide further evidence that STRAP stabilizes β-catenin through GSK-3β signaling and the effect of Wnt signaling in activating β-catenin is stronger than that of STRAP. Interestingly, even β-catenin expression was restored by Wnt3a or GSK-3β inhibitors, treated cells with STRAP knockdown still showed less tumorigenic properties when compared with control cells (Figure 3D and 3E). This indicates that STRAP can promote tumor cell growth and invasion not only through Wnt/β-catenin pathway, but may also through regulating other signaling pathways, such as TGF-β signaling and MAPK pathway [31, 32]. From this study it is difficult to distinguish the effect of STRAP on tumor growth and metastasis *in vivo*. However, our *in vitro* studies indicate that downregulation of STRAP in CRC cells inhibit cell migration and invasion as shown in Figure 1 and Supplementary Figure S1. STRAP knockdown stable clones showed lower cell invasion (Figure 3E) when compared with vector control and parental cells with the same treatments with GSK-3β inhibitors and Wnt3a, suggesting that downregulation of STRAP in CRC cell lines inhibits cell migration and invasion, at least in part, directly through inhibiting Wnt/β-catenin signaling.

In this study, we also found that APC truncation at 1338aa, which loses all the Axin binding domains but still have one 20 amino acid repeat for β-catenin binding, partially compromise the effect of STRAP stabilizing β-catenin. While an activating phosphorylation site mutation in the N-terminus of β-catenin that stabilizes it, completely supplanted the effect of STRAP (Figure 6B and 6C). In contrast, STRAP stabilizes β-catenin in colon cancer cell lines with wild type APC and β-catenin. It is possible that the effect of the mutations in APC and β-catenin in stabilizing β-catenin is stronger than that of STRAP.

We observed in 130 colorectal cancer specimens that STRAP is upregulated in about 70% cases (Figure 7C and Supplementary Table S2). Interestingly, we found that the expression of both STRAP and β-catenin were much higher in AJCC stage I than that in other stages. These, coupled with the finding that STRAP is also upregulated in 50.8% colorectal adenomas [28], indicate that both STRAP and β-catenin function in the early stage of CRC. Besides, there is highly significant correlation between the expression of STRAP and β-catenin including its nuclear accumulation in these tumor samples (Figure 7B, 62.5% vs 41.3%). These observations indicate that STRAP can interact with other proteins and regulate their function to regulate CRC development and progression.

In summary, these studies demonstrate novel mechanistic insights into the functions of STRAP in colorectal cancer invasion and metastasis. STRAP decreases the phosphorylation, and increases stabilization of β-catenin through interaction with GSK-3β. Thus, STRAP promotes CRC initiation and progression through activating Wnt/β-catenin signaling by inhibiting β-catenin ubiquitin-dependent degradation and nuclear localization (Figure 7D). This study provides a rationale for targeting STRAP for therapeutic intervention in colorectal cancers.

## MATERIALS AND METHODS

### Cell culture

Mouse colon adenocarcinoma cell lines MC38 and CT26; human colon adenocarcinoma cell lines SW480, SW620, HCT116 and RKO; non-small cell lung cancer (NSCLC) cell lines A549 and H460; and HEK-293T cells were maintained in 7% serum-containing medium supplemented with penicillin and streptomycin.

### Plasmids

HA-tagged or Flag-tagged STRAP (in pCDNA3 vector) has been described previously [11]. Myc-tagged GSK-3β (in pJ3M vector) and HA-tagged β-catenin (in pCDNA3 vector) were gifts from Dr. Alan Diehl (University of Pennsylvania Cancer Center) and Dr. Stephen Byers (Georgetown University School of Medicine, WA), respectively. The His_6_-Ubiquitin expression construct was a gift from Dr. Christoph Eglert (Leibniz Institute for Age Research, Jena, Germany). TOP/FOP Flash reporter constructs were gifts from Dr. Eric Fearon (University of Michigan Medical School, Ann Arbor, MI).

### Regents and antibodies

Proteasomal inhibitor MG132 and GSK3β inhibitor SB415286 were purchased from Selleckchem.com and TOCRIS bioscience (Bristol, UK), respectively. Lithium chloride was obtained from Calbiochem (La Jolla, CA). Cycloheximide was purchased from Sigma (St. Louis, MO). Human and mouse Wnt3a was purchased from R & D Systems (Minneapolis, MN). Antibodies were purchased as follows: anti-CyclinD1, anti-HA, anti-c-Myc, anti-RhoA, and anti-poly (ADP) ribose polymerase (PARP) from Santa Cruz Biotechnology (Santa Cruz, CA); anti-STRAP and anti-β-catenin from BD Transduction Labs (San Jose, CA); anti-phospho-β-catenin (Ser33/37/Thr41), anti-GSK-3β, anti-APC and anti-phospho-GSK-3β (S9) from Cell Signaling Technology (Danvers, MA); and anti-β-actin and anti-Flag antibodies from Sigma (St. Louis, MO).

### Stable STRAP knockdown cell lines

Cell lines (as indicated) were infected with STRAP shRNA lentivirus and selected with puromycin. STRAP knockdown polyclonal populations of SW480, HCT116, and RKO were generated using similar protocol. The expression of STRAP was verified by western blotting.

### Western blot and immunoprecipitation analyses

Western blot and immunoprecipitation analyses were performed as previously described [11]. For subcellular localizations of endogenous β-catenin, nuclear and cytoplasmic protein extracts were prepared as previously described [40]. Lysates were analyzed by western blotting as indicated in figure legends. In order to investigate the effect of STRAP on the β-catenin binding to the degradation complex, 293T cells were transfected with expression constructs. After 48 h, cells were harvested for immunoprecipitation which has been described previously [11]. Then the immunoprecipitates were analyzed by western blotting

### Cell counting assays

MC38 and CT26 cells were plated in 12-well plates. After 48 h, cells were counted every day for 5 days and the average cell numbers from triplicate wells were plotted.

### Soft agarose assays and xenograft studies

MC38 and CT26 cells were plated for soft agarose assays as described previously [40]. For xenograft studies, 1 × 10^5^ cells from STRAP knockdown stable clones and vector control cells derived from MC38 and CT26 cell lines were subcutaneously injected into C57BL/6 and Balb/c mice, respectively. All animal experiments were performed in accordance with IACUC and state and federal guidelines for the humane treatment and care of laboratory animals. The animals were monitored for tumor formation every 3 days for a total of 3–5 weeks and the tumors were measured as discussed previously [41].

### Migration and invasion assays

Migration and invasion assays were performed as previously described [41]. MC38 and CT26 cells were allowed to migrate for 6 hours, and to invade for 12 hours through collagen and for 21 hours through matrigel. After migration or invasion, cells were fixed, stained, and counted from 6 random fields and averaged.

### Real time qRT-PCR

Total RNA was extracted from MC38, CT26, H460 and A549 cells using Trizol reagent and RT-PCR amplification was performed using iScript^™^ Reverse Transcription Supermix (BIO-RAD, Hercules, CA). Real-time PCR was carried out using 2.5 ul cDNA with FastStart SYBR Green Master (Roche, Nutley, NJ) following the manufacturer's instruction. The primer sequences of STRAP, β-catenin, β-TrCP, MMP2, MMP7, MMP9 and GAPDH were shown in Supplementary Table S1.

### TOP/FOP flash reporter assay

Cells were seeded in 12-well plates. After overnight incubation, TOP/FOP Flash reporter plasmid (0.5 ug/well) were transfected into cells with Lipofectamine^2000^ following the manufacturer's protocol. β-galactosidase (25 ng/well) was used as a control for the transfection efficiency. After approximately 44 hours, cells were harvested and luciferase assays were performed using Monolight^™^ 3010 (BD Pharmingen, San Diego, CA) according to the manufacturer's protocol. Transfection of each group was performed in triplicate. Luciferase reading values were normalized with β-gal values and triplicates were averaged.

### Gelatin zymography

For gelatin zymography, cells were seeded in 6-well plates. After overnight incubation, cells were treated with 900 ul serum-free media for another 16 hours and the media were collected. After normalized with total protein of the cell lysate, the media were subjected to zymography analyses with non-reduced SDS-PAGE using 10% gels containing 0.1% gelatin according to the protocol as discussed elsewhere [42].

### *In vivo* ubiquitination assay

HEK-293T cells were transiently transfected with combinations of expression constructs his_6_-ubiquitin, β-catenin-HA and STRAP-Flag with lipofectamine^2000^ (Invitrogen, Calsbad, CA). Forty hours after transfection, the cells were treated with 25 μM of MG132 for 6 hours. Then the cells were lysed in highly denaturing conditions with 6 M guanidine hydrochloride buffer and ubiquitylated proteins were pulled down from the lysates with Ni–NTA agarose resin following the manufacturer's protocol. The bound proteins were eluted with 2X Laemmli buffer containing 250 mM imidazole. The eluants were analyzed by western blotting.

### Splenic injection and orthotopic cecum injection models

Splenic injection and cecum injection models were described in our previous reports [41, 43]. Briefly, for splenic injection, 1 × 10^5^ cells in 100ul PBS were injected into the spleens. 5 min after injection, spleens were removed. C57BL/6 mice injected with MC38 cells were sacrificed after 3 weeks, whereas Balb/c mice injected with CT26 cells were sacrificed after 4 weeks for analysis of tumor formation in the liver. For cecum injection, 1 × 10^5^ LM10 cells, derived from MC38 cells, were suspended in 50 ul PBS and were injected subserosally into ceca using a 30-G needle under stereomicroscope. Mice were monitored and sacrificed 48 days after injection when some mice became moribund. The tumors in ceca, livers, and lymph nodes were examined for primary and metastatic tumor growth.

### Immunohistochemical analyses

Paraffin-embeded mouse xenograft tissue and human colon cancer tissue microarray slides were subjected to immunostaining. The slides were stained as we described previously [41]. Anti-STRAP antibody at 1:50 and anti-β-catenin antibody at 1:300 dilutions were used for staining. The immunohistochemical evaluation for human colon cancer tissues for the expression of STRAP and β-catenin was according to that previously described, including the determination of staining intensity and the percentage of cells stained [3, 44, 45]. The proportion score represents the estimated fraction of positive cells (0 = 0%, 1 = 1%–24%, 2 = 25%–49%, 3 = 50%–74%, and 4 = 75%–100%), while the intensity score represents their average staining intensity (0 = no staining; 1 = weak staining, 2 = moderate staining, 3 = strong staining). The final staining score was determined by multiplying the intensity score by the proportion score. As a result, scoring was between 0 and 12. “Up-regulation” means that the score for the cancer tissues is higher than the score for the matched normal tissues. “no-change” means that the score for the cancer tissues is equal to the score for the matched normal tissues. “Down-regulation” means that the score for the cancer tissues is lower than the score for the matched normal tissues. When evaluating the expression of STRAP, we defined a score of 0–4 as low and 6–12 as high, respectively. Regarding the localization of β-catenin, we tried to divide the staining pattern into four groups according to The Human Protein ATLAS's β-catenin expression in CRC (http://www.proteinatlas.org/ENSG00000168036-CTNNB1): cytoplasmic/membranous/nuclear, cytoplasmic/membranous, nuclear, and none.

### Statistical analysis

The data are presented as mean ± S.D. Statistical analyses were performed by Student's *t*-test or analysis of variance (ANOVA) with Bonferroni post hoc test. Spearman rank correlation coefficient analysis was performed to analyze the correlation between STRAP and β-catenin expression in human CRC samples. Chi-square was used to analyze β-catenin nuclear accumulation in different STRAP expression groups in human CRC samples. Kruskal-Wallis test was used to analyze the associations between the expression of STRAP or β-catenin and clinical information, including AJCC stage, pathology grade and age. Statistical analysis was performed with SPSS software for windows (version 16.0; SPSS, Chicago, IL). A 2-sided *p* value of less than .05 was considered statistically significant.

## SUPPLEMENTARY MATERIALS FIGURES AND TABLES


