# Supplementary material for: Novel role of STRAP in progression and metastasis of colorectal cancer through Wnt/β-catenin signaling
Source: Oncotarget. 2016 Feb 20;7(13):16023–37. doi: 10.18632/oncotarget.7532 (PMC4941295; doi:10.18632/oncotarget.7532)
Supplement: Supplementary file 1 [file oncotarget-07-16023-s001.pdf]

Novel role of STRAP in progression and metastasis of colorectal cancer through Wnt/β-catenin signaling

Supplementary Materials

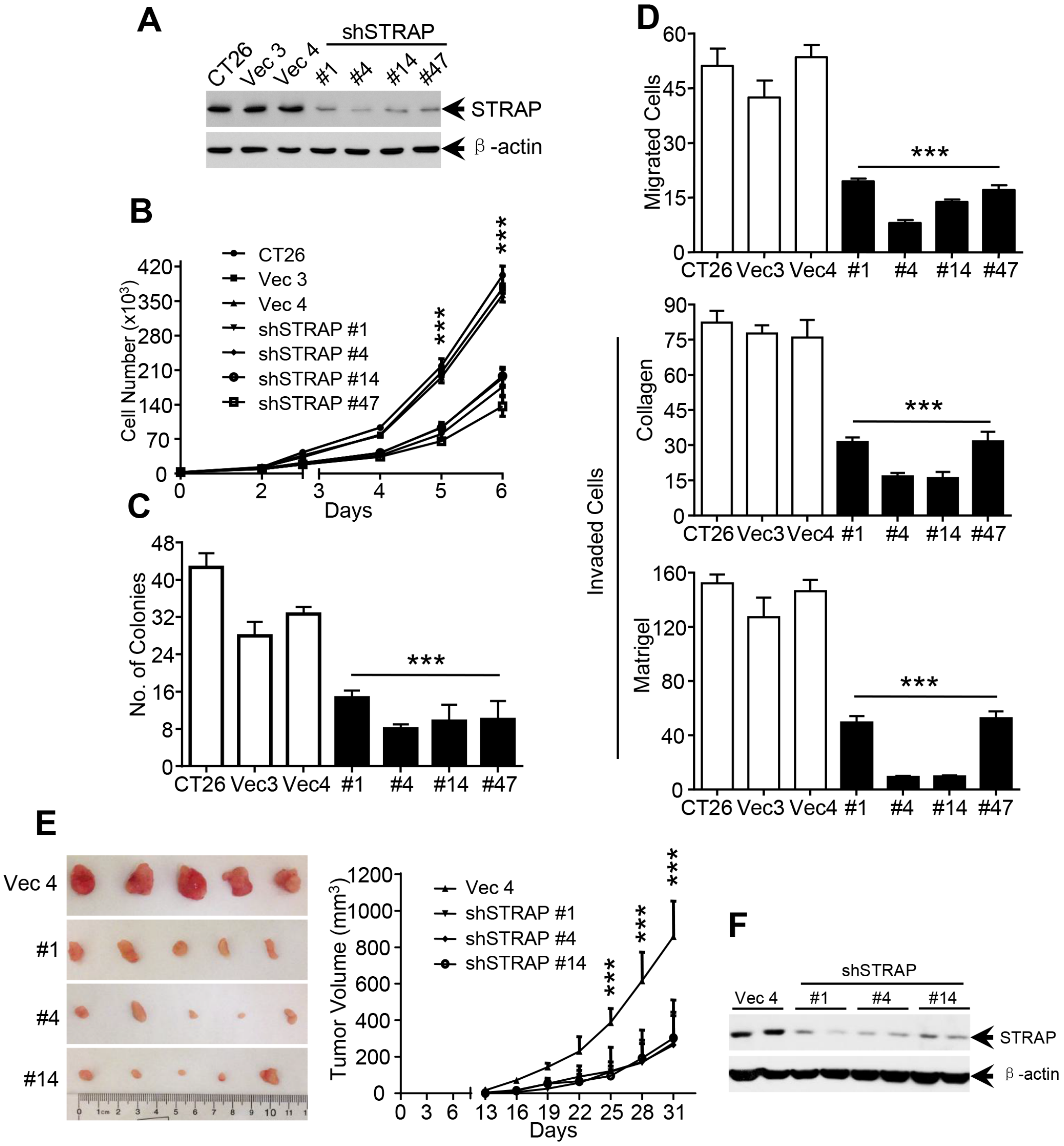

Supplementary Figure S1: Effect of downregulation of STRAP on migration, invasion and tumorigenicity in CRC cell line. (**A**) Expression of STRAP in CT26 stable clones with STRAP shRNA was examined by western blotting. β-actin was used as loading control. (**B**) Cell counting assay. CT26 stable clones with STRAP knockdown and parental and control vector cells were cultured in RPMI 1640 containing 7% FBS for a total of 6 days. Cells were counted everyday for 5 days from the third day after the cells were seeded and the cell numbers are plotted. Individual data points are mean ± S.D. of triplicate determinations. ANOVA with Post Hoc Tests was used to determine statistical significance. Error bars represent standard deviation. ****P* < .001. (**C**) Soft agarose assay. CT26 cells were cultured in 0.4% sea plague agarose for 12 days. Number of colonies is counted and shown as mean ± S.D. of triplicate wells. ANOVA, ****P* < .001. (**D**) Cell migration and invasion assay. CT26 cells were allowed to migrate through transwells (top), and pass through a collagen barrier (middle) or a matrigel layer (bottom) in the transwell chambers. Six random high power fields in each well were counted. Each data point represents mean ± S.D. from three wells. ANOVA, ****P* < .001. (**E**) Suppression of tumorigenicity of CT26 in vivo by knockdown of STRAP. Macroscopic subcutaneous tumors from mice injected with vector control and three STRAP knockdown clones are shown (left). Results are presented as mean ± S.D. of the tumor volume (right). ANOVA, ****P* < .001. (**F**) The expression of STRAP in subcutaneous tumors was analyzed by western blotting.

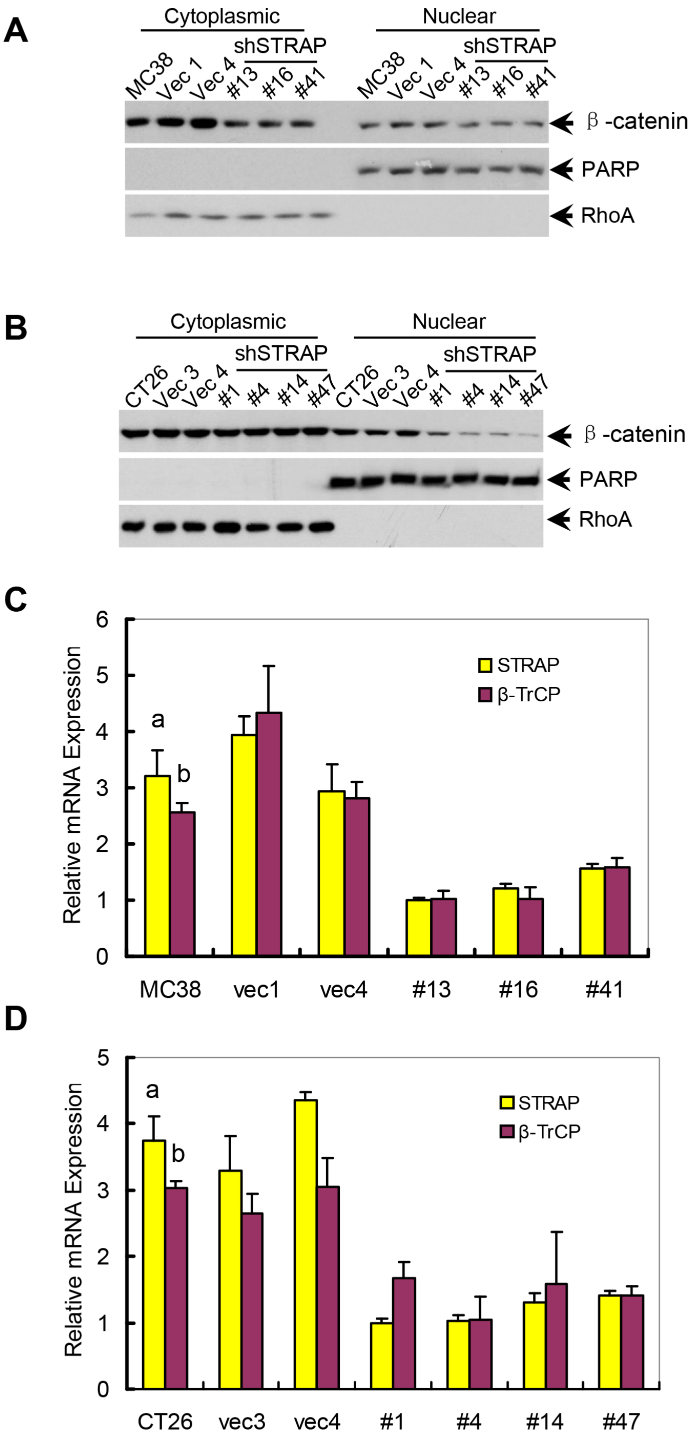

Supplementary Figure S2: Role of STRAP on regulating β-catenin expression in the cytoplasm and nucleus of CRC cell lines. (**A**) and (**B**) The expression of β-catenin protein in cytoplasmic and nuclear compartments of MC38 (A) and CT26 (B) stable clones with STRAP downregulation was detected by western blotting. Complete fractionations of cytoplasmic and nuclear proteins were verified by western blotting with antibodies against RhoA and PARP. C & D. The expression of STRAP and β-catenin signaling downstream target gene β-TrCP in MC38 (**C**) and CT26 (**D**) was analyzed by real time qRT-PCR. a & b, P <.001. All compares among parental, vector and shSTRAP groups.

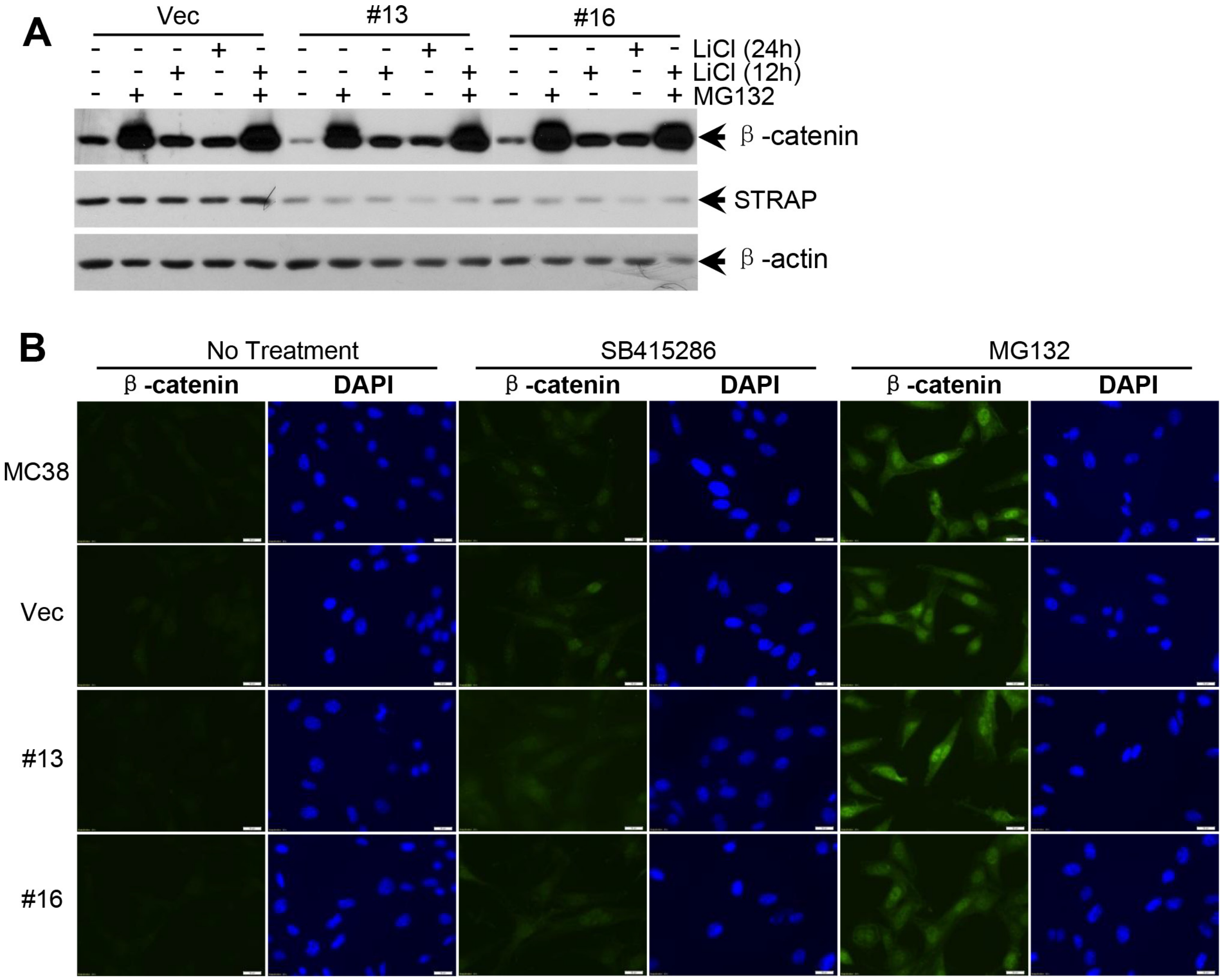

Supplementary Figure S3: Role of GSK-3β inhibitors and proteasomal inhibitor on STRAP-induced stabilization of β-catenin. (**A**) MC38 stable clones were treated with GSK-3β inhibitor LiCl (20 mM, two time points) and proteasomal inhibitor MG132. Then the lysates were subjected to western blotting β-catenin and STRAP. (**B**) Subcellular distribution of β-catenin in MC38 cells was analyzed by immunofluorescence staining after treated with SB415286 or MG132.

A

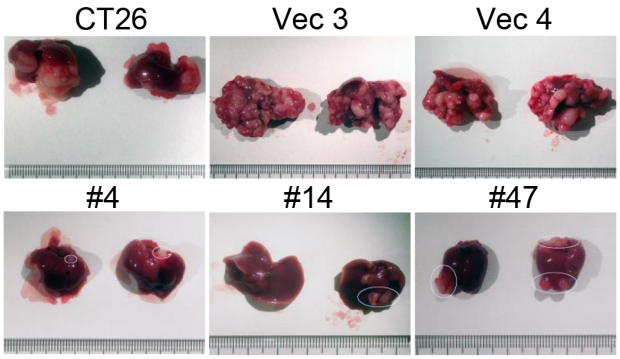

B

The effect of STRAP knockdown on inhibition of liver metastasis

Mice	# of mice	Total wt (mean±S.D.)	Liver wt *** (mean±S.D.)
CT26	5	19.81 (1.58)	3.16 (0.76)
Vec 3	4	18.43 (1.19)	3.67 (0.79)
Vec 4	3	17.95 (1.52)	3.58 (0.52)
#1	5	19.86 (1.48)	1.15 (0.24)
#4	5	20.17 (1.27)	1.06 (0.15)
#14	5	19.59 (0.90)	1.19 (0.26)
#47	5	20.36 (1.04)	1.20 (0.12)

C

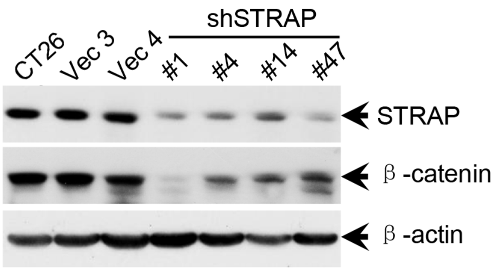

D

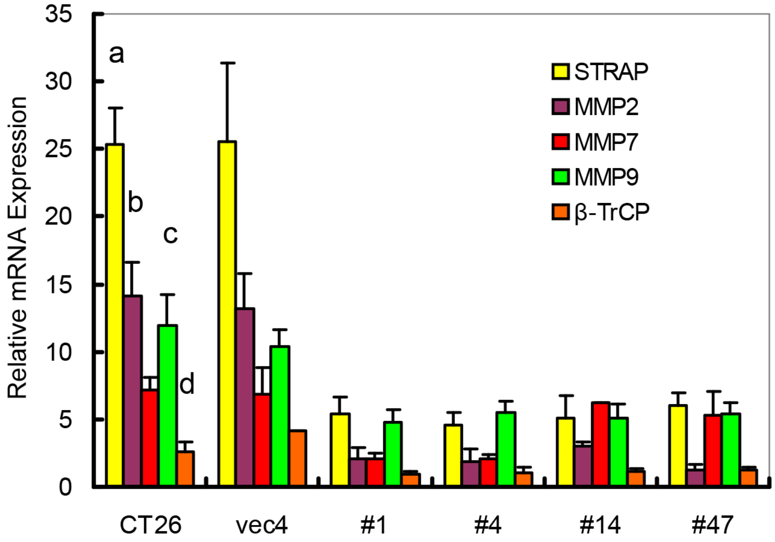

Supplementary Figure S4: Role of STRAP on CRC metastasis in a splenic injection model. (**A**) and (**C**) CT26 stable clones were injected into spleens of Balb/c mice (*n* = 5, in each group). Five weeks after injection the mice were sacrificed. Representative pictures of liver metastasis were shown and the foci of liver metastases and liver weights were assessed. ANOVA with Post Hoc Tests was used to determine statistical significance. Standard deviations were included. ***$P < .001$. (**B**) The expression of STRAP and β-catenin in the lysates from liver metastasis were analyzed by western blotting. (**D**) The expression of β-catenin signaling downstream target genes β-TrCP, MMP2, MMP7 and MMP9 in the primary tumor tissues was analyzed by real time qRT-PCR. a, b ,c & d $P < .001$, all compares among parental, vector and shSTRAP groups.

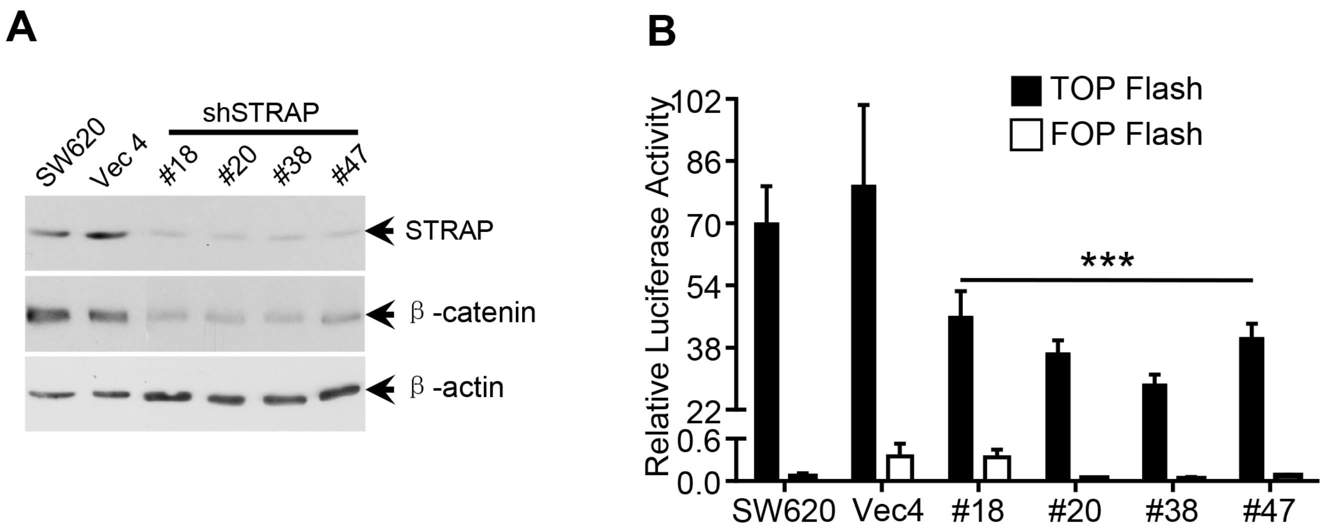

Supplementary Figure S5: Role of APC truncation on STRAP induced stabilization of β-catenin. (**A**) Expression of STRAP and β-catenin in SW620 stable clones with STRAP shRNA was examined by western blotting. β-actin was used as loading control. (**B**) The transcriptional activity of Wnt/β-catenin signaling was detected in SW620 STRAP knockdown stable clones using TOP/FOP Flash reporter as described above. ANOVA with Post Hoc Tests was used to determine statistical significance. Error bars represent standard deviation. ***$P < .001$.

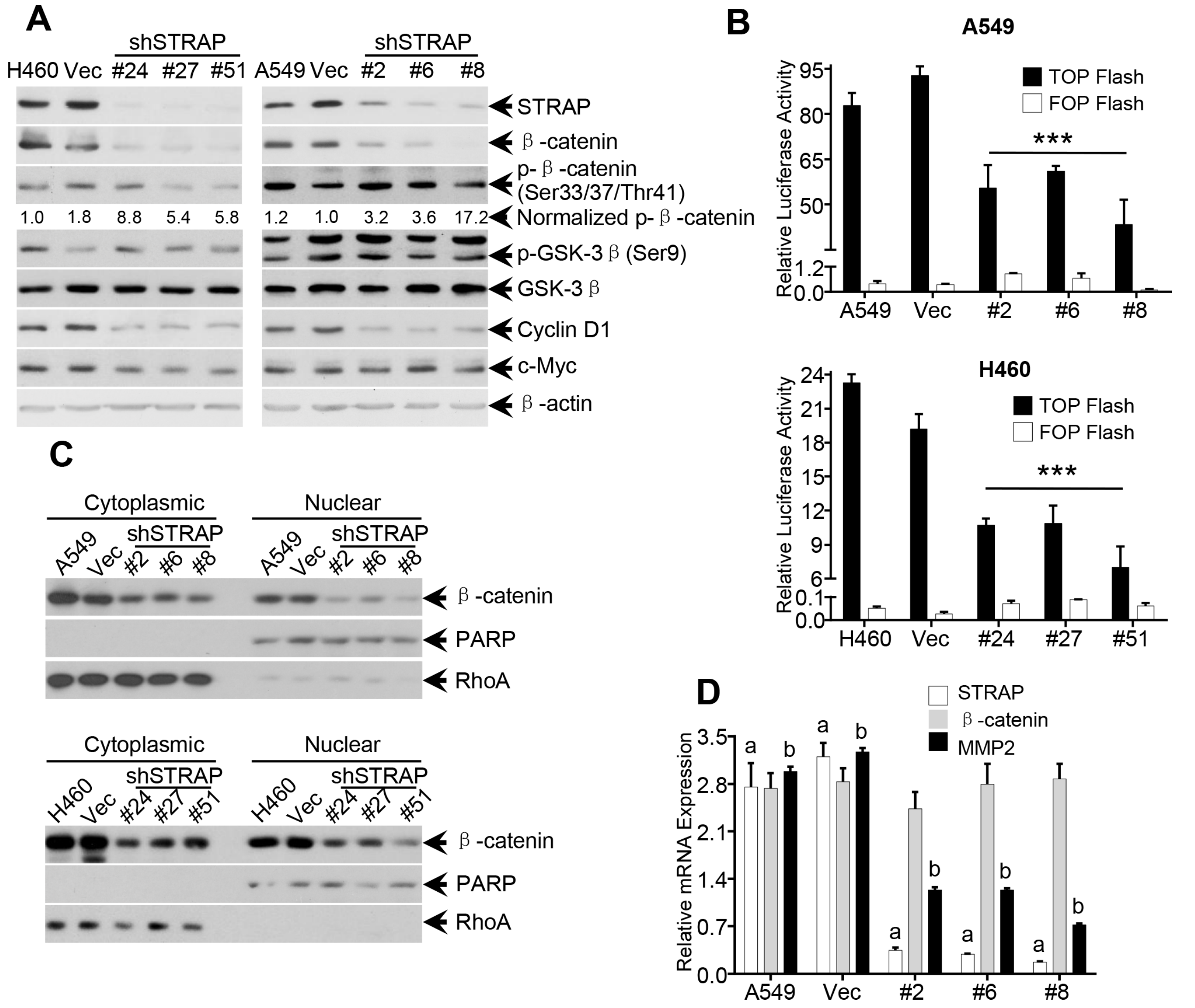

Supplementary Figure S6: Activation of Wnt/β-catenin signaling through its stabilization by STRAP in lung cancer cell lines. (**A**) Some important components of Wnt/β-catenin signaling were detected by western blotting with the lysates from NSCLC cell lines A549 and H460 after STRAP knockdown. (**B**) The transcriptional activity induced by Wnt/β-catenin signaling was inhibited by knockdown of STRAP in both A549 and H460 cell clones. ANOVA with Post Hoc Tests was used to determine statistical significance. Error bars represent standard deviation. ***$P < .001$. (**C**) Both cytoplasmic and nuclear β-catenin was downregulated in A549 and H460 STRAP knockdown stable clones. Complete fractionations of cytoplasmic and nuclear proteins were verified by western blotting with antibodies against RhoA and PARP. (**D**) The expression of β-catenin, STRAP and MMP2 was detected by real time PCR as detailed in the Materials and Methods. ANOVA, a & b, $P < .001$.

Supplementary Table S1: Primer sequences used for Real-Time qRT-PCR analyses

Primers		Sequence	Annealing temperature (°C)	Product Size (bp)
GAPDH	Forward	TGTGAACGGATTTGGCCGTA	57.0	224
	Reverse	GGTCTCGCTCCTGGAAGATG	57.4	
STRAP	Forward	GGATGCGGTCTCAGGAGATG	57.4	208
	Reverse	ATCGTCACTGCACCACAGAG	57.2	
β-catenin	Forward	ACTTGCCACACGTGCAATTC	56.7	165
	Reverse	ATGGTGCGTACAATGGCAGA	57.1	
β-TrCP	Forward	AAGATAGT CAGCGGCCTT C	57.0	259
	Reverse	ACAGCGATGGAACGGTCTT	57.0	
MMP2	Forward	CCCCCGATGCTGATACTGAC	57.5	263
	Reverse	CACAGCCTTCTCCTCCTGTG	57.6	
MMP7	Forward	GGTGTGGAGTGCCAGATGTT	60.2	231
	Reverse	GATCTCTCCTTGCGAAGCCA	59.8	
MMP9	Forward	TCTGCCTGCACCACTAAAGG	57.4	288
	Reverse	CAGGCTGTACCCTTGGTCTG	57.6	

Supplementary Table S2: The expression of STRAP and β-catenin in CRC specimens

Variables	STRAP (*n*, %)	P-catenin (*n*, %)
Up-regulation	100 (76.9)	84 (71.2)
No change	19 (14.6)	33 (28.0)
Down-regulation	11 (8.5)	1 (0.8)

Details of staining and scoring were described in Supplementary Methods.
